# Vaccine-Induced Antibodies Mediate Higher Antibody-Dependent Cellular Cytotoxicity After Interleukin-15 Pretreatment of Natural Killer Effector Cells

**DOI:** 10.3389/fimmu.2019.02741

**Published:** 2019-11-27

**Authors:** Leigh Fisher, Melissa Zinter, Sherry Stanfield-Oakley, Lindsay N. Carpp, R. Whitney Edwards, Thomas Denny, Zoe Moodie, Fatima Laher, Linda-Gail Bekker, M. Juliana McElrath, Peter B. Gilbert, Lawrence Corey, Georgia Tomaras, Justin Pollara, Guido Ferrari

**Affiliations:** ^1^Vaccine and Infectious Disease Division, Fred Hutchinson Cancer Research Center, Seattle, WA, United States; ^2^Department of Surgery, Duke University Medical Center, Durham, NC, United States; ^3^Duke University Medical Center, Duke Human Vaccine Institute, Durham, NC, United States; ^4^Perinatal HIV Research Unit, Faculty of Health Sciences, University of the Witwatersrand, Soweto, South Africa; ^5^The Desmond Tutu HIV Centre, University of Cape Town, Cape Town, South Africa; ^6^Department of Biostatistics, University of Washington, Seattle, WA, United States; ^7^Department of Immunology, Duke University Medical Center, Durham, NC, United States; ^8^Department of Molecular Genetics and Microbiology, Duke University Medical Center, Durham, NC, United States

**Keywords:** antibody-dependent cellular cytotoxicity, HIV vaccine trial, interleukin-15, natural killer cells, HIV-1 infectious molecular clone-infected target cell assay

## Abstract

The secondary analyses for correlates of risk of infection in the RV144 HIV-1 vaccine trial implicated vaccine-induced antibody-dependent cellular cytotoxicity (ADCC) responses in the observed protection, highlighting the importance of assessing such responses in ongoing and future HIV-1 vaccine trials. However, *in vitro* assays that detect ADCC activity in plasma from HIV-1 infected seropositive individuals are not always effective at detecting ADCC activity in plasma from HIV-1 vaccine recipients. *In vivo*, ADCC-mediating antibodies must operate at the site of infection, where effector cells are recruited and activated by a local milieu of chemokines and cytokines. Based on previous findings that interleukin 15 (IL-15) secretion increases during acute HIV-1 infection and enhances NK cell-mediated cytotoxicity, we hypothesized that IL-15 pretreatment of NK effector cells could be used to improve killing of infected cells by vaccine-induced antibodies capable of mediating ADCC. Using the HIV-1 infectious molecular clone (IMC)-infected target cell assay along with plasma samples from HIV-1 vaccine recipients, we found that IL-15 treatment of effector cells improved the ability of the vaccine-induced antibodies to recruit effector cells for ADCC. Through immunophenotyping experiments, we showed that this improved killing was likely due to IL-15 mediated activation of NK effector cells and higher intracellular levels of perforin and granzyme B in the IL-15 pretreated NK cells. We also found that using a 4-fold dilution series of plasma and subtraction of pre-vaccination responses resulted in lowest response rates among placebo recipients and significant separation between treatment groups. This represents the first attempt to utilize IL-15-treated effector cells and optimized analytical approaches to improve the detection of HIV-1 vaccine-induced ADCC responses and will inform analyses of future HIV vaccine clinical trials.

## Introduction

Antibody-dependent cellular cytotoxicity (ADCC) is an immune mechanism that bridges the adaptive humoral and innate immune responses. ADCC has important applications in cancer treatment, via monoclonal antibody (mAb)-mediated ADCC killing of tumor cells (1), and is also involved in host defense from viral infection and control of viremia, via ADCC-mediated killing of virus-infected cells (2). In the latter, antibodies (Abs) bind pathogen antigens expressed on the membrane of infected cells, which can then recruit and activate Fc-gamma receptor (FcγR)-expressing cytotoxic effector cells to kill the infected cells (3). Classic ADCC is driven by natural killer (NK) cells expressing FcγRIIIa (CD16A), which binds to the Fc region of IgG Abs recognizing virus infected target cells (4). An increasing body of evidence suggests that ADCC contributes to protection against HIV-1 acquisition in pre-clinical studies (5–9); importantly, the immune correlates analysis of the RV144 HIV-1 efficacy trial of the ALVAC-HIV canarypox vector with the gp120 AIDSVAX B/E vaccine found that, in vaccine recipients with low levels of anti-Env IgA Abs, increased ADCC activity was associated with lower risk of HIV-1 acquisition (10). This finding, combined with later follow up work (11–13), supports the hypothesis that the protection afforded by the RV144 vaccine regimen was due in part to ADCC activity of vaccine-induced Abs (14).

Within the HIV Vaccine Trials Network (HVTN), two ADCC assays have been qualified according to GCLP guidelines and are currently used to assess ADCC activity induced by candidate vaccines: the gp120-coated target cell assay (15) and the HIV-1 infectious molecular clone (IMC)-infected target cell assay (hereafter referred to as the Luc assay) (13). As assessed by the gp120-coated target cell assay, ADCC activity has been shown to correlate with control of viremia (5, 7, 8), protection from challenge (9) in pre-clinical studies in rhesus macaque models of HIV infection, and prevention of mother-to-infant transmission in clinical studies (6). The Luc assay is a modified version of the ADCC assay used in the primary correlates analysis for RV144 (10) and has been shown to reliably detect ADCC responses in serum samples from HIV-1 infected individuals (16), ADCC responses in plasma samples from RV144 vaccine recipients (17), and ADCC mediated by mAbs isolated from RV144 vaccine recipients (13, 17, 18). Interestingly, the Luc assay was not able to detect ADCC responses in plasma samples from AIDSVAX B/B vaccine recipients in the VAX004 trial at significantly higher levels than in placebo recipients [(16) and G. Ferrari, unpublished observations]. The Luc assays were performed using cryopreserved effector cells from HIV-seronegative donors after overnight rest in absence of any *in vitro* activation and expansion of the effector cell subsets. We hypothesized that vaccine-induced Abs capable of mediating ADCC against infected target cells in the Luc assay may have not been able to adequately engage the resting NK cells. Therefore, we explored the possibility of improving NK cell performance without a long-term *in vitro* expansion step.

It has long been known that interleukin-15 (IL-15) activates NK cells (19–21). Moreover, we have previously demonstrated that IL-15 can increase the ability of NK cells to engage ADCC-mediating Abs induced by natural infection in a more specific manner than IL-12, IL-18, or IL-21 (22). There is other evidence that IL-15 may help improve NK cell performance in the Luc assay: depletion experiments have demonstrated that IL-15 is important for maintaining the homeostasis of NK cell subsets (23, 24), and IL-15 has been proposed to have a critical role in the development (25–27) and education (28, 29) of NK cells. It has also been reported that IL-15-receptor-α (IL-15Rα) is essential for activating and increasing cytotoxic activity and interferon-gamma (IFN-γ) production by NK cells (30). Lastly, IL-15 has been proposed to be involved in the homing of NK cells (31). Cumulatively, these observations demonstrate that IL-15 is a key regulator of the development, maturation, survival, activation, and migration of NK cells.

We hypothesized that vaccine-induced Abs capable of ADCC might be more efficient in recruiting IL-15-treated NK cells, as these cells better represent those that traffic to the site of infection compared to their non-IL-15-treated counterparts. Using plasma samples from vaccine recipients receiving an ALVAC prime, protein boost regimen and placebo recipients, we tested this hypothesis by pre-incubating the effector cells with IL-15 in the Luc assay to detect vaccine-induced ADCC responses. Our results indicated that ADCC-mediating Abs were more efficient in recruiting effector cells upon their incubation with IL-15 in absence of proliferative activity and/or strong upregulation of activation markers.

## Materials and Methods

### HVTN 100

The HVTN 100 phase 1/2 trial (ClinicalTrials.gov identifier NCT02404311) was a randomized, controlled, double-blind trial performed in South Africa (32). Adults (18–40 years) were allocated to the vaccine regimen or placebo at a 5:1 ratio. The vaccine regimen in Part A consisted of ALVAC-HIV administered at months 0, 1, 3, 6, and 12, along with MF59-adjuvanted bivalent subtype C gp120 administered at months 3, 6, and 12. The primary outcomes of the trial have been described previously (32).

### Study Samples

Assays were run on 40 blinded plasma samples from a subset of HVTN 100 trial participants, consisting of *n* = 34 vaccine and *n* = 6 placebo recipients. For each selected participant, plasma samples were available for both the baseline visit and the visit 2 weeks after the fourth vaccination (at month 6.5). The subset was randomly selected from the per-protocol (those who had received the first 4 vaccinations) participants with plasma samples available from both visits.

Human PBMC samples, collected by leukapheresis procedure, and HIV-1 seronegative and seropositive plasmas were collected in accordance with protocols approved by the Duke University Institutional Review Board. Signed written informed consent was received from study participants for the use of anonymized samples for research purposes prior to inclusion in this study. The HVTN 100 trial was approved by the research ethics committees of the University of the Witwatersrand, the University of Cape Town, the University of KwaZulu-Natal, and the Medical Research Council; all participants gave written informed consent in English or in their local language (Setswana, Sotho, Xhosa, or Zulu).

### Laboratory Methods

#### Phenotypic Characterization of Natural Killer (NK) Cells

Immunophenotyping of human NK cells was performed using flow cytometry analyses. Cryopreserved peripheral blood mononuclear cells (PBMC) collected from 15 healthy normal adult donors were thawed and incubated overnight (18 h) in RPMI 1640 medium supplemented with 10% FBS, or in RPMI 1640 medium supplemented with 10% FBS and 10 ng/mL recombinant human IL-15 (Miltenyi Biotec, GmbH). The cells were then washed with PBS and stained with a viability marker (Fixable Aqua Dead Cell Stain Kit, Thermo Fisher Scientific, San Diego, CA) prior to surface and intracellular staining with fluorescently conjugated Abs using standard techniques. The staining panel used to identify NK cell subsets and phenotypes was based on the Optimized Multicolor Immunofluorescence Panel (OMIP) described previously [OMIP-007, (33)]. Fluorescently conjugated Abs used for surface staining were: PE-TR-CD3, clone S4.1, Thermo Fisher Scientific; APC-H7-CD4, clone SK3, BD Biosciences, San Jose, CA; PE-Cy5-CD14, clone Tuk4, Thermo Fisher Scientific; PE-Cy5-CD19, clone SJ25-C1, Thermo Fisher Scientific; PacificBlue-CD16, clone 3G8, BD Biosciences; PE-Cy7-CD56, clone NCAM16.2, BD Biosciences; BV606-CD62L, clone DREG-56, Biolegend, San Diego, CA; FITC-HLA-DR, clone G46-6, BD Biosciences; APC-CD57, clone HCD57, Biolegend; and BV785-CD69, clone FN50, Biolegend. Intracellular staining was performed with BV711-Perforin, clone dG9, Biolegend, and PE-Granzyme B, clone GB11, BD Biosciences. Quantum™ Simply Cellular^®^ beads (Bangs Laboratories, Inc., Fishers, Indiana) were used to determine the Ab binding capacity (ABC) of perforin and granzyme within cells according to the manufacturer's recommended procedure. Data analysis was performed using FlowJo software (v10.5.3).

#### Infectious Molecular Clones (IMCs)

A replication-competent IMC, similar to that described in Pollara et al. (13), was used to generate the HIV-1 reporter virus. Three constructs were used, encoding the *env* gene for subtype AE CM235 (GenBank Accession No. AF259954.1; plasmid provided by Dr. Jerome Kim, US Military HIV Research Program), subtype C 1086.c (GenBank Accession No. FJ444395), or subtype C TV-1 (GenBank Accession No. HM215437), in addition to the *Renilla* luciferase reporter gene (34, 35). Transfection of 293-T cells with proviral IMC plasmid DNA yielded the three Env-IMC-LucR reporter viruses, hereafter referred to as NL-LucR.T2A-AE.CM235-ecto (IMC_CM235_), C.1086.c (IMC_1086.c_), and C.TV-1 (IMC_TV−1_). Reporter virus stocks were generated as described in Pollara et al. (13) and infection titer quantified by infection of TZM-bl target cells as described in Li et al. (34).

#### Infection of Cultured CEM.NKR_CCR5_ Cells With HIV-1 IMCs

A total of 1 × 10^6^ CEM.NKR_CCR5_ cells [NIH AIDS Reagent Program, Division of AIDS, NIAID, NIH from Dr. Trkola (36)] were infected with a TCID_50_ per cell of 0.069 for IMC_CM235_, 71.4 for IMC_1086.c_, and 10.5 and 1.4 TCID_50_ per cell for IMC_TV−1_ by incubation for 0.5 h at 37°C and 5% CO_2_ in the presence of DEAE-dextran (7.5 μg/ml). These amounts of virus were determined using our titration of the stock to achieve a frequency of p24+ (i.e., IMC-infected) cells >10% and viability of target cells >40%. The cells were subsequently resuspended at 0.5 × 10^6^/ml and cultured for 48 to 72 h in complete medium containing 7.5 μg/ml DEAE-dextran. For each ADCC assay, we monitored the frequency of infected target cells by flow cytometry intracellular p24 staining.

#### Infected Cell ADCC Assay

We utilized a modified version of the ADCC luciferase assay (13). Briefly, CEM.NKR_CCR5_ cells were infected with HIV-1 IMCs as described above and used as target cells. For effector cells, cryopreserved PBMC obtained by leukapheresis from a HIV-seronegative individual (Fc-gamma-Receptor IIIa 158 V/F phenotype) were thawed the day before the assay and rested overnight in RPMI 1640 medium supplemented with antibiotics and 10% fetal bovine plasma (R10), with or without recombinant human IL-15 at a concentration of 10 ng/ml. Effector, represented by whole PBMC, and target cells (30:1 ratio) were plated in opaque 96-well half-area plates and co-cultured with serial dilutions of plasma. Each plasma sample was assayed at six dilutions, starting at a dilution of 1:50, with duplicate wells set up for each dilution. For the 4-fold serial dilution scheme, plasma dilutions of 1:50, 1:200, 1:800, 1:3200, 1:12800, and 1:51200 were used. Co-cultures were incubated for 6 h at 37°C in 5% CO_2_, either with recombinant human IL-15 (10 ng/ml) or without. The assay readout is luminescence intensity (measured in relative light units, RLUs) generated by surviving target cells that have not been lysed by the effector population in the presence of ADCC-mediating plasma Abs. The mAb palivizumab (Synagis), which mediates ADCC (37) but is specific for respiratory syncytial virus, and a cocktail of HIV-1 mAbs (HIV-1 mAb mix) demonstrated to mediate ADCC [A32 (38), 2G12 (39), CH44 (40), and 7B2 (41)] were used as negative and positive controls, respectively. The HIV-specific mAbs were produced using recombinant techniques, and were generated using a human IgG1 constant region containing alanine substitutions (S298A, E333A, K334A) designed to enhance binding to Fcc-receptor IIIa (FccR3A) (42).

### Statistical Methods

The RLU intensity of the wells containing only target and effector cells (no plasma) represents spontaneous lysis in the absence of any Abs. These wells were used as control wells for calculating non-specific background activity. Percent specific killing was calculated according to the following formula: 100 × (Average RLU in target plus effector alone (no Abs or plasma) wells—Average RLU in experimental wells)/(Average RLU in target plus effector alone (no Abs or plasma) wells). Two different types of dilution curves were analyzed: (1) unadjusted percent killing dilution curves of baseline and post-vaccination plasma samples; and (2) baseline-subtracted percent killing dilution curves, where positive baseline values were subtracted from post-vaccination percent killing values at each dilution. For both the unadjusted and baseline-subtracted dilution curves, the peak percent (%) killing was defined as the maximum activity across the six dilution levels (“peak % killing” or “peak baseline-subtracted % killing”). A response was defined as positive if the peak baseline-subtracted % killing was greater than or equal to 10% in one of the first two dilutions. Although the peak is used to define positivity, baseline-subtracted dilution curves were also summarized with a non-parametric partial area under the baseline-subtracted curves (pAUCs), calculated using the trapezoidal rule on the first four dilutions of the baseline-subtracted curves, setting baseline-subtracted killing <0% to zero. Further details of these methods can be found in the Supplementary Material.

Bar charts were used to display positive response rates and boxplots show the distribution of responses. Two-sided 95% confidence intervals (95% CI) for positive response rates were computed via the Wilson score method (43). Comparisons of response rates and magnitudes between samples run against the same IMC with IL-15 pretreated and untreated effector cells used McNemar's exact text and the Wilcoxon signed rank test for paired data and considered significant if the two-sided *p*-value was < 0.05. The Benjamini and Hochberg was used to adjust for multiple comparisons where note. All analyses were performed in R 3.3.3 (44).

## Results

### IL-15 Improves Detection of ADCC Responses in the Luc Assay When Using Plasma Samples From HIV-1 Infected Seropositive Individuals

**Figure 1:**
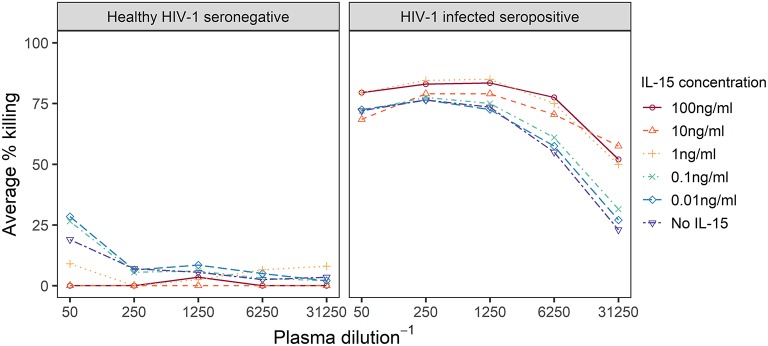
Dilution curves representing observed % specific killing in ADCC assays performed with effector cells treated overnight with IL-15 at the indicated concentrations and plasma from a healthy HIV-1 seronegative or a HIV-1 infected seropositive individual. IL-15 concentration levels below 1 ng/ml are varying shades of blue, while higher concentrations are in oranges and reds.

We first explored the effect of the IL-15 concentration used to pretreat effector cells in the Luc assay with plasma samples from one HIV-1 infected seropositive and one healthy HIV-1 seronegative individual. Effector cells were treated overnight with IL-15 concentrations ranging from 0 to 100 ng/ml IL-15, and then used to assay each plasma sample; ADCC activity against IMC_CM235_-infected target cells detected for the six dilutions of IL-15 is summarized in Figure 1 (Results from additional dilutions and independent replicates are shown in the Supplementary Material). When the assay was performed with the seronegative sample, the lowest levels of ADCC activity were observed when the effector cells were pretreated with more than 1 ng/mL IL-15. Specifically, no ADCC activity was detected when effector cells were pretreated with 10 ng/ml IL-15. Furthermore, when the assay was performed with the HIV-1 infected seropositive sample, the highest levels of ADCC activity were observed when the effector cells were pretreated with more than 1 ng/mL IL-15, especially at the higher plasma dilutions. Overall, 10 ng/mL IL-15 was the lowest concentration of IL-15 that minimized non-specific ADCC activity while simultaneously improving specific ADCC activity when compared to assays performed with untreated effector cells using this screening procedure. This conclusion is further supported by our published data using a panel of NK cells isolated from HIV-1 seronegative (*n* = 12) and seropositive (*n* = 6) subjects (22). This observation suggested that IL-15 pretreatment of effector cells may improve the detection of ADCC responses in plasma samples from HIV-1 vaccine recipients.

### IL-15 Also Improves Detection of ADCC Responses in the Luc Assay When Using Well-Characterized ADCC-Mediating mAbs Specific for HIV-1 Infected Cells

**Figure 2:**
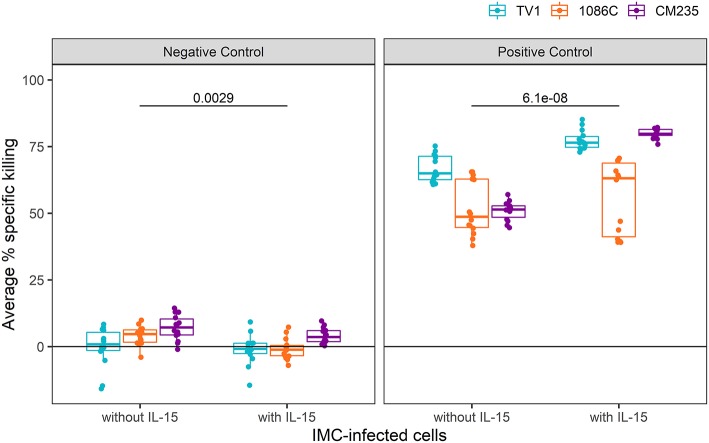
Average % killing in Synagis negative control wells **(Left)** and HIV-1 mAb mix positive **(Right)** control wells, shown by IMC. Each dot represents the average of duplicate control wells per experimental plate (*N* = 14). Unadjusted Wilcoxon *p*-values comparing responses with and without IL-15 are reported.

We next examined whether similar improvements were obtained with IL-15-pretreated effector cells when using a cocktail of well-characterized mAbs known to mediate ADCC against HIV-1 infected cells (positive control wells) in comparison to a well-characterized mAb that also mediates ADCC but is specific for respiratory syncytial virus-infected cells (Synagis; negative control wells). As some non-neutralizing antibodies that mediate ADCC have been found to have relatively narrow breadth of HIV-1 Env recognition (45), we performed assays using target cells infected with IMCs representing three different HIV-1 Env isolates. This approach allowed us to also assess whether the enhancing effects of IL-15 on measured ADCC activity were independent of the specific HIV-Env used in the assay. For each IMC, 28 replicates from 14 independent experiments were run with and without IL-15-pretreated NK cells (10 ng/ml); the observed responses are summarized as average percent killing, following the formula described in section statistical methods and plotted in Figure 2. The combined responses from all Synagis negative control wells were significantly lower when effector cells were pretreated with IL-15 compared to when untreated were used, with the median response more than 5-fold higher in plates without IL-15 (Wilcoxon *p*-value = 0.003). The combined responses among all HIV-1 mAb mix positive control wells had 1.4-fold higher median response with the addition of IL-15 compared to without IL-15 (Wilcoxon *p*-value < 0.001). Of note, these effects were observed even though the Fc region of the mAbs was already optimized for binding to the Fc-γR IIIa as reported in the methods. Similar trends were observed for IMC-specific responses. The median IMC-specific responses in Synagis negative control wells were between 0.8 and 7.1% without IL-15 and between −1.2 and 3.6% in the presence of IL-15. For HIV-1 mAb mix positive control wells, the median IMC-specific responses were between 49 and 65% in the absence of IL-15 and between 63 to 80% with pretreated effector cells. With all three envelope targets, lower average percent specific killing was observed when effector cells pretreated with IL-15 were used compared to untreated cells in the lab negative control, and higher average percent specific killing was observed when effector cells pretreated with IL-15 were used compared to untreated cells in the lab positive control (Figure 2). Together, these results indicate that the pretreatment of effector cells with IL-15 reduces the background and results in higher responses for target cells infected with all IMCs tested; albeit, the extent to which IL-15 increases the response magnitude and reduces response variability appears to have some antigen-related variability.

### Impact of IL-15 on NK Cellular Subsets, Activation State, and Cytotoxic Effector Molecule Content

**Figure 3:**
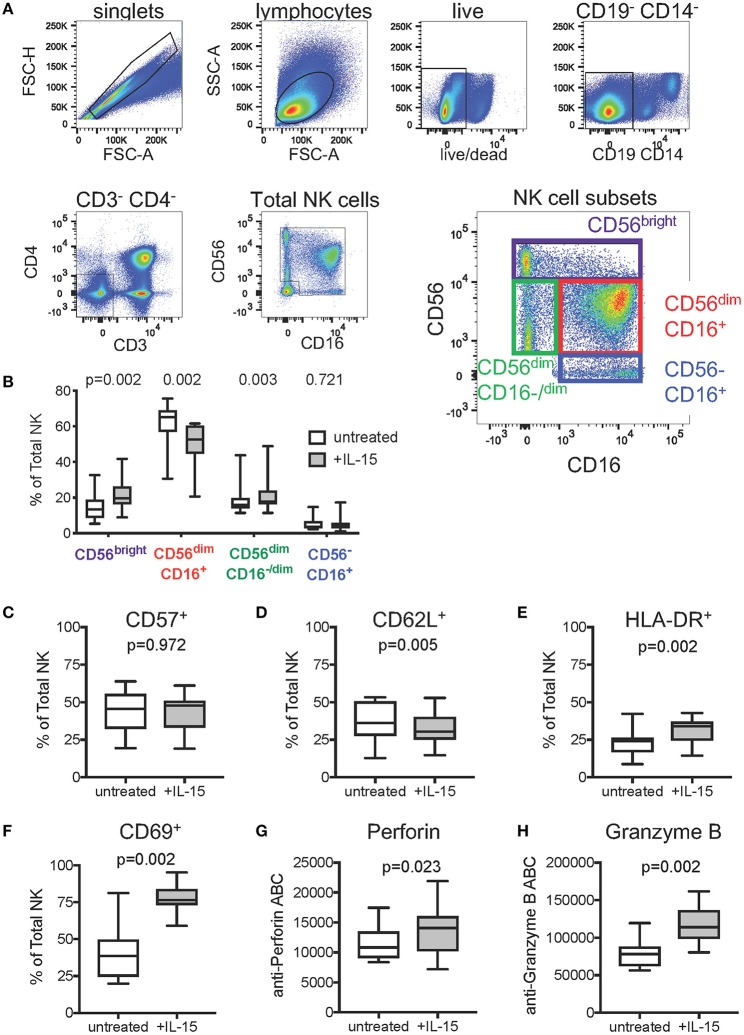
Effect of exogenous IL-15 treatment on phenotypes of natural killer (NK) cells. **(A)** Gating strategy used to identify total NK cells and NK cell subsets. Concatenated data representing all samples (*n* = 30, 15 PBMC donors ± IL-15) was included in the flow plots. **(B)** Comparison of NK cell subset frequencies when untreated, and after overnight treatment with 10 ng/ml IL-15 (*n* = 15 paired PBMC samples per condition). Frequencies of total NK cells expressing **(C)** CD57, **(D)** CD62L, **(E)** HLA-DR, and **(F)** CD69 in cells left untreated or treated overnight with 10 ng/ml IL-15. Amount of intracellular **(G)** perforin and **(H)** Granzyme B in NK cells left untreated or after overnight treatment with 10 ng/ml IL-15, represented as antibody binding capacity (ABC), which is determined from the mean fluorescence intensity (see Methods). In **(B–H)**, box plots represent the interquartile ranges, horizontal lines indicate the medians, and error bars extend to the minimum and maximum observed values. *P*-values were calculated with paired Wilcoxon rank sum tests and were adjusted for multiple comparisons to control for false discovery rate via the Benjamini-Hochberg method.

To gain mechanistic insight into our findings that IL-15 pretreatment of effector cells reduces background and enhances the levels of ADCC detected in the Luc assay, we used flow cytometric immunophenotyping to examine the effect of IL-15 treatment on natural killer cell subset frequencies, activation state, and cytotoxic effector molecule content. Prior to staining, PBMC from 15 healthy adult human donors were incubated overnight in cell culture medium, or medium supplemented with 10 ng/ml recombinant human IL-15. The gating strategy used to identify total NK cells and NK cell subsets is indicated in Figure 3A. As shown in Figure 3B, we observed significant differences in the distribution of major NK cell subsets (46–49) with paired Wilcoxon rank sum tests after IL-15 treatment—increased frequency of CD56^bright^ NK cells (*p* = 0.002), decreased CD56^dim^CD16^+^ NK cells (*p* = 0.002), and increased CD56^dim^CD16^−/dim^ NK cells (*p* = 0.003). No significant differences were observed for the frequency of CD56^−^CD16^+^ NK cells, a subset that is predominantly functionally impaired (48). We saw no difference in the frequency of total NK cells that expressed the maturation marker CD57 (Figure 3C), however, treatment with IL-15 did result in reduced expression of the secondary lymphoid homing marker CD62L (*p* = 0.005, Figure 3D). NK cells incubated with IL-15 presented a more active phenotype than untreated NK cells as evidenced by significantly higher surface expression of HLA-DR (*p* = 0.002, Figure 3E) and CD69 (*p* = 0.002, Figure 3F) (50, 51). We next used fluorescent quantitation beads to measure the intracellular content of perforin and granzyme B to determine if IL-15 treatment affected the abundance of these cytotoxic effector molecules. We found that NK cells treated with IL-15 had significantly more intracellular perforin (Figure 3G) and granzyme B (Figure 3H) compared to untreated cells.

### Baseline Plasma Samples

**Figure 4:**
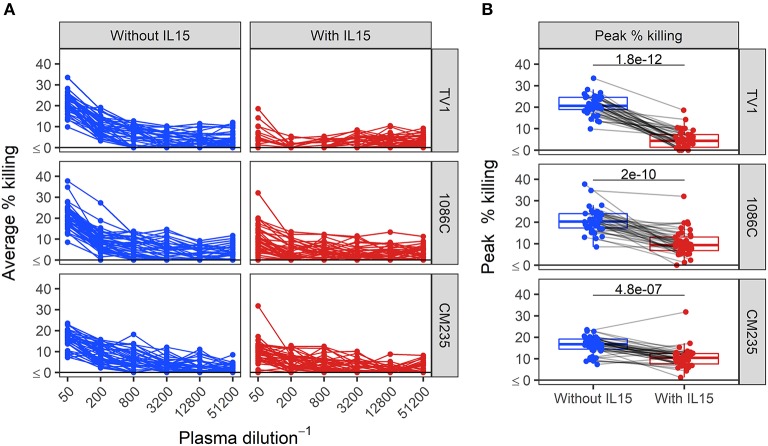
Dilution curves **(A)** and boxplots of peak % killing **(B)** of baseline ADCC activity in baseline plasma samples from HVTN 100 participants, with and without IL-15. Unadjusted *p*-values from paired Wilcoxon rank sum test are reported. Responses from the same sample are connected with gray lines.

We next assessed ADCC activity of baseline samples from an HIV vaccine trial (HVTN 100), where all participants were HIV-1 negative at enrollment and baseline samples should lack HIV-specific ADCC activity. We performed Luc assays at serial plasma dilution schemes ranging from 2 to 4-fold. The results, shown in the Supplementary Material, indicated that we could not reach endpoint concentration with the 2-fold and the 3-fold dilution schemes in 80 and 40% of positive samples, whereas the 4-fold dilution scheme provided endpoint titers in 100% of positive samples from vaccine recipients. Dilution curves in baseline samples from HVTN 100 participants and boxplots summarizing peak responses from baseline samples are presented in Figure 4. When effector cells are pretreated with IL-15, the dilution curves tended to be flatter compared to those obtained by assays performed using untreated cells (Figure 4A). Median peak % killing was consistently lower when IL-15 was included in the assay, across IMCs (Figure 4B). For IMC_TV1_-, IMC_1086.C_-, and IMC_CM235_-infected target cells, peak % killing in the presence of IL-15 was significantly lower than in its absence (paired Wilcoxon *p*-values < 0.0001 for each IMC), with median fold drop in magnitude of 0.21-, 0.50-, and 0.62-fold lower using IL-15 treated effector cells, respectively.

### Baseline-Subtraction to Account for Non-specific ADCC

**Figure 5:**
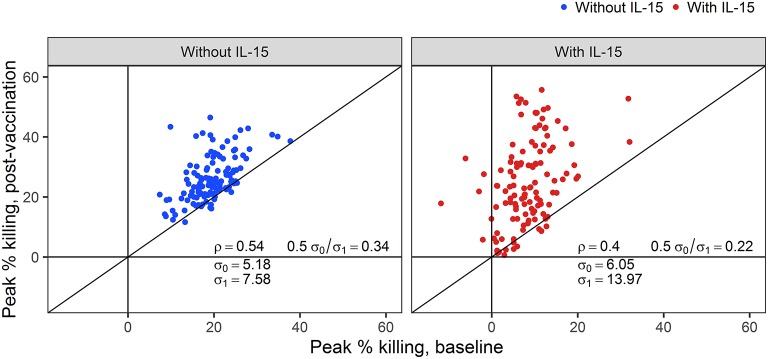
Correlations (ρ) between post-vaccination and pre-vaccination ADCC responses with and without NK cells pretreated with IL-15. The standard deviation for baseline (post-vaccination) observations are denoted by σ_0_ (σ_1_).

While the addition of IL-15 reduced the peak % killing in baseline samples, ADCC activity above 15% killing was still detectable at the 1:50 dilution in 18% of samples tested against IMC_1086.C_-infected cells. In the context of vaccine trials, where vaccine-induced ADCC activity is of primary interest, adjusting post-vaccination outcomes to account for differences in baseline ADCC activity is reasonable, and under certain conditions may improve the statistical properties of the outcome (52). To evaluate the utility of baseline-subtraction, post-vaccination ADCC responses are plotted against baseline ADCC responses in Figure 5. Correlations between baseline and post-vaccination responses are sufficiently high to further support the use of baseline subtraction. Hence, for the remainder of this manuscript, we focus on baseline-subtracted ADCC responses.

### Baseline-Subtracted ADCC Activity

**Figure 6:**
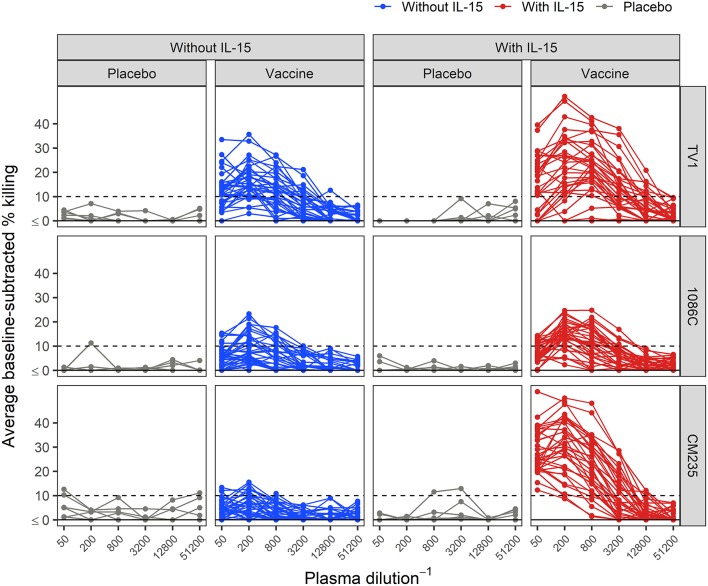
Average baseline-subtracted % killing curves for placebo and vaccine recipients, in the presence and absence of IL-15. Target cells were infected with the TV1, 1086C, or CM235 IMC. Results from placebo samples are shown in gray. The black dashed line is at 10%, the positivity threshold for baseline-subtracted % killing.

**Figure 7:**
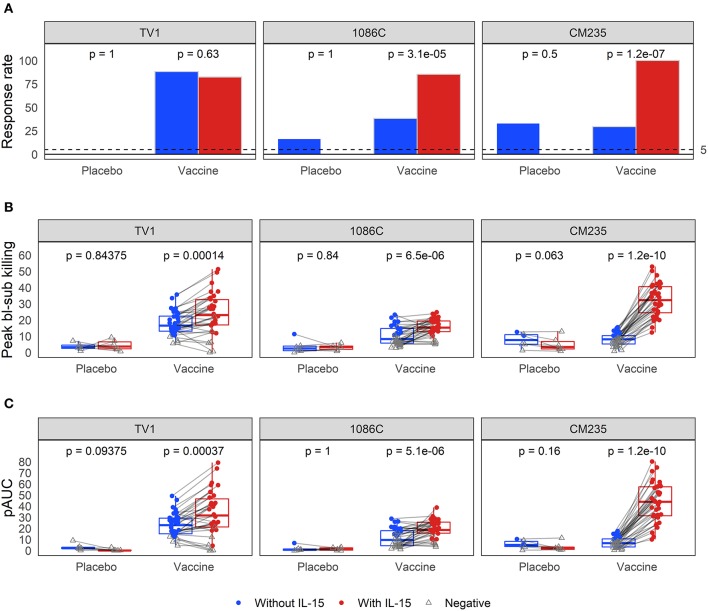
Comparison of **(A)** response rates, **(B)** peak % killing, and **(C)** pAUCS as determined from the baseline-subtracted ADCC response curves, performed with the three different IMCs in the presence and absence of IL-15. Responses from the same sample are connected with gray lines. McNemar *p*-values are reported for differences in response rates; paired Wilcoxon *p*-values are reported for both peak % killing and pAUC. All *p*-values are unadjusted. TV1 and 1086C are both vaccine-matched, while CM235 is not.

Baseline-subtracted dilution curves for ADCC activity are shown in Figure 6. Response rates, peak responses, and pAUC values of baseline-subtracted dilution curves from assays performed with or without IL-15 are shown in Figure 7. The assays were performed with target cells infected with one of each of the three IMCs. In the presence of IL-15, the response rate among the 6 placebo recipients was 0% (95% CI: 0%−39%, computed via the Wilson score method) for all three IMCs and consistently lower than that observed in the absence of IL-15; however, these differences are not statistically significant (Figure 7A). Response rates of vaccine recipients are higher in the presence of IL-15 for all three IMC-infected cells, although not statistically different for IMC_TV1_-infected cells. For IMC_1086.C_-infected cells, response rates among vaccine recipients were 38% (Wilson 95% CI: 24–055%) in the absence of IL-15 compared to 85% (Wilson 95% CI: 70–94%) in the presence of IL-15, with McNemar *p*-value < 0.001. For IMC_CM235_-infected cells, response rates among vaccine recipients were 29% (Wilson 95% CI: 17–46%) in the absence of IL-15 compared to 100% (Wilson 95% CI: 90–100%) in the presence of IL-15, with McNemar *p*-value < 0.001. It should be noted that the samples were batch-tested in both conditions against every IMC to exclude interference from inter-assay variability. The magnitudes of peak baseline-subtracted responses among vaccine recipients were significantly higher in the presence of IL-15 than those observed in the absence of IL-15 for all three IMCs tested, with paired Wilcoxon *p*-values < 0.001 for each (Figure 7B). For cells infected with vaccine-matched IMCs (IMC_TV1_ and IMC_1086.C_), the median fold change was 1.39-, 1.46-fold higher in the presence of IL-15. For IMC_CM235_-infected cells, the median fold change was 3.6-fold higher with the addition of IL-15. Among placebo recipients, peak baseline-subtracted responses were similar between the two assay conditions. Analysis of the pAUC for each of the IMC-infected target cells with and without IL-15 revealed similar results (Figure 7C).

## Discussion

In this study, we demonstrated that detection of ADCC-mediated killing of HIV-1 infected cells by the Luc assay is improved when the effector populations are exposed to IL-15 during overnight incubation prior to the assay. This observation held true when testing plasma from one HIV-1 infected seropositive individual, well-characterized mAbs known to mediate ADCC, and plasma samples from HIV-1 vaccine recipients. Given that vaccine-induced ADCC has been shown to correlate with lower risk of HIV-1 infection in vaccine recipients with low anti-Env IgA responses (10), our finding that IL-15 augments ADCC responses has implications for how this model could replicate the *in vivo* response that has been linked with protection. Interestingly, one study found that in 90% of individuals with acute HIV-1 infection, plasma IL-15 levels rose rapidly as viremia increased, reaching significantly elevated concentrations in the pg/mL of plasma range compared to their baseline (i.e., when the HIV-1 viral load first became detectable) levels (53). This finding would suggest that NK cells could be rapidly recruited to the site of infection as previously postulated (54), similar to what has been reported in a mouse model of influenza infection (55). However, a complete understanding of how plasma IL-15 concentrations extrapolate to tissue sites of infection remains unknown. Moreover, IL-15 signaling primarily works by trans-presentation, where the IL-15 is bound to the IL-15Rα on the surface of a presenting cell, and signals by forming a complex with the IL-15β/γC on responding cells (56). Therefore, we do not know the extent to which the *in vitro* concentration used in this study is representative of tissue concentration and/or presentation by IL-15Rα-bearing cells *in vivo*. Despite this limitation, our data suggest that exposure to IL-15, either in the circulation or in tissue sites of infection, likely results in a higher level of activation of mucosal NK cells that is expected to promote increased ADCC-mediated killing of HIV-1 infected cells. This hypothesis could be investigated in future work.

Our immunophenotyping experiments shed light on the potential mechanism by which IL-15 augments ADCC responses. Compared to their non-IL-15-pretreated counterparts, IL-15-pretreated NK cells displayed significant increases in the frequency of cytokine-secreting CD56^bright^ (47) and polyfunctional CD56^dim^CD16^−/dim^ cells (49). However, neither of these increases are expected to contribute to the improvement in ADCC activity observed after IL-15 treatment as most cells in these subsets lack expression of the CD16 Fc receptor needed for canonical ADCC. The NK cell populations observed to be increasing were offset by a reduction in the frequency of conventional CD56^dim^CD16^+^ NK cells (46), with no change in the more functionally impaired CD56^−^CD16^+^ subset (48). These findings suggest that IL-15 pre-treatment may promote a balanced NK cell response. Consistent with prior studies, we also found a reduction in the lymphoid homing marker CD62L on IL-15-pretreated NK cells. *In vivo*, this reduction would likely facilitate NK cell egress from secondary lymphoid tissue (57, 58). Importantly, we saw no difference in the frequency of total NK cells that expressed cell-surface CD57, suggesting that the short duration of IL-15 treatment used in our study did not change the maturation state or proliferative potential of the NK cells (59). However, we did observe that IL-15-pretreated NK cells expressed higher levels of HLA-DR and CD69, indicating that these cells were in a more activated state (50, 51), and that they had higher intracellular levels of perforin and granzyme B, thus providing an explanation for the increased cytotoxic potential of the IL-15-pretreated cells as observed in the Luc assay. We do not currently have an explanation for the reduced background observed in the presence of IL-15; further studies will be performed to understand the reason for this observation.

In the context of HVTN trials, where we are trying to elicit an ADCC response in seronegative individuals, the Luc assay was not sensitive enough to distinguish moderate vaccine-induced responses from assay noise. Strict positivity criteria were necessary to control the false positive rate, which suppressed the response rate among vaccine recipients. Following our previous statistical optimization of pilot ADCC data (10), this represents one of the first times a formal approach has been taken to optimize readouts for detecting vaccine-induced ADCC activity. From a technical point of view, incubation of IL-15-treated effector cells with the optimized dilution scheme in combination with baseline subtraction improved performance of the Luc assay, and supports the use of these modifications in future analyses of vaccine-induced ADCC activity in HIV vaccine trials. The value in having a full toolbox of assays for quantitating ADCC activity was demonstrated by Huang et al. (16), whose findings indicated that, although the results obtained by different ADCC assays were generally similar to each other, they still exhibited variation, presumably due to the technical differences between the assays (population-level readout vs. single cell-readout, whole PBMC as the effector population vs. an NK cell-enriched effector population, etc.). The HIV-1 Luc assay utilized in this study is a modification of the assay previously described by Alpert et al. (60). We have used this assay to demonstrate crucial synergies between V1V2 mAbs representative of the V1V2 Ab responses correlated with low risk of infection in RV144 and C1C2 mAb responses representing the immunodominant ADCC responses in the RV144 clinical trial (13). The IMC virus used for infection contains a luciferase reporter gene that has been described to interfere with expression of Nef-dependent CD4 downregulation (61), thereby promoting a CD4-induced conformation. However, we have previously shown that cells infected with these viruses do downregulate CD4, likely due to the activity of the Vpu protein, and a large portion of the envelope on the surface is not accessible by an antibody specific to the CD4-induced conformation (62). Thus, use of these infected target cells provides a broad assessment of the potential Env conformations that can serve as targets for ADCC antibodies including CD4-induced, closed trimer, and the recently defined stage 2A — a more open CD4-induced conformation that has been described as an important target for vaccine strategies (63) and has been associated with control of HIV-1 replication in a report of linked HIV-1 transmission (64).

We note that the idea of using cytokines to activate NK cells and thereby enhance ADCC activity has long been explored in the context of cancer immunotherapy with mAbs, starting with the finding that incubation of peripheral blood lymphocytes with IL-2 activated a subset of these lymphocytes (referred to as lymphokine-activated killer cells, or LAKs) that includes NK cells (65). The approach of using LAKs in combination with mAbs appeared to show promise in clinical trials, with the addition of LAKs augmenting ADCC in patients with B-cell lymphoma (66). While IL-15 was also thought to have promise in this context, its short half-life *in vivo* long limited its applications in cancer immunotherapy (67). Recent advancements in improving the *in vivo* half-life and efficacy of IL-15 have generated new interest in IL-15 cancer immunotherapies that combine optimized versions of IL-15 with ADCC-mediating mAbs that target cancer cells (67, 68). IL-15 has been shown to promote NK cell activity in human *ex vivo* models of mAb-based therapies for chronic lymphocytic leukemia, leading to improved leukemic B cell depletion, and to improve the therapeutic efficacy of mAb-based therapy for colon cancer in mice. In the context of HIV-1 infection, IL-15 has been proposed as an adjuvant in DNA vaccine regimens (69) as well as an immune therapeutic component of strategies aimed at eliminating the reservoir of latently HIV-1 infected cells (22).

The data collected in this study suggest that ADCC responses could potentially be underestimated using resting effector cells, a finding with implications for all ADCC assays, not just the Luc assay as examined here.

## Data Availability Statement

The datasets generated for this study are available on request to the corresponding author.

## Ethics Statement

The studies involving human participants were reviewed and approved by Fred Hutchinson Cancer Research Center IRB. Duke University IRB. The patients/participants provided their written informed consent to participate in this study.

## Author Contributions

LF analyzed the data. MZ, SS-O, RE, and JP designed and performed the experiments. LF, LNC, JP, and GF interpreted the data. TD collected and characterized the leukapheresis samples. PG, LC, JP, and GF conceived and supervised the project. LF, LNC, JP, and GF wrote the manuscript. FL, L-GB, and ZM contributed to HVTN 100 design and data collection. LF, MZ, SS-O, LNC, RE, TD, ZM, FL, L-GB, MM, PG, LC, GT, JP, and GF commented on the manuscript and approved the final version.

### Conflict of Interest

The authors declare that the research was conducted in the absence of any commercial or financial relationships that could be construed as a potential conflict of interest.
