# Supplementary material for: Vaccine-Induced Antibodies Mediate Higher Antibody-Dependent Cellular Cytotoxicity After Interleukin-15 Pretreatment of Natural Killer Effector Cells
Source: Front Immunol. 2019 Nov 27;10:2741. doi: 10.3389/fimmu.2019.02741 (PMC6890556; doi:10.3389/fimmu.2019.02741)
Supplement: Supplementary file 1 [file Presentation_1.pdf]

SUPPLEMENTARY MATERIAL

for "Vaccine-Induced Antibodies Mediate Higher Antibody-Dependent Cellular Cytotoxicity After Interleukin-15 Pretreatment of Natural Killer Effector Cells"

by Fisher and Zinter et al.

Statistical methods

Positivity

A response was defined as positive if the peak baseline-subtracted % killing was greater than or equal to 10% in one of the first two dilutions. The positivity definition was derived to primarily to control the false positive rate among placebo recipients, while accounting for the fact that response curves in vaccine recipients are often non-monotonic, due to a prozone effect. Together, this suggested that if there was detectable ADCC activity, we would expect to see some signal in the most concentrated dilutions, even if the peak activity is detected at lower concentrations. As responses in negative control wells did not exceed 10% in any sample assayed with IL-15 pretreated NK cells (Figure 2 in the primary manuscript), we set the positivity threshold to 10%. In Figure S1 we plot baseline-subtracted ADCC dilution curves by the dilution at which the peak response is observed and by vaccination group (note this is the IL-15-pretreated data in Figure 6 of the primary manuscript). Among placebo recipients, ADCC responses rarely exceeded 10%. In the one curve that does exceed 10%, it does so at the 1:800 dilution and reaches its peak at the 1:3200 dilution, however the first two dilutions have no detectable ADCC activity.

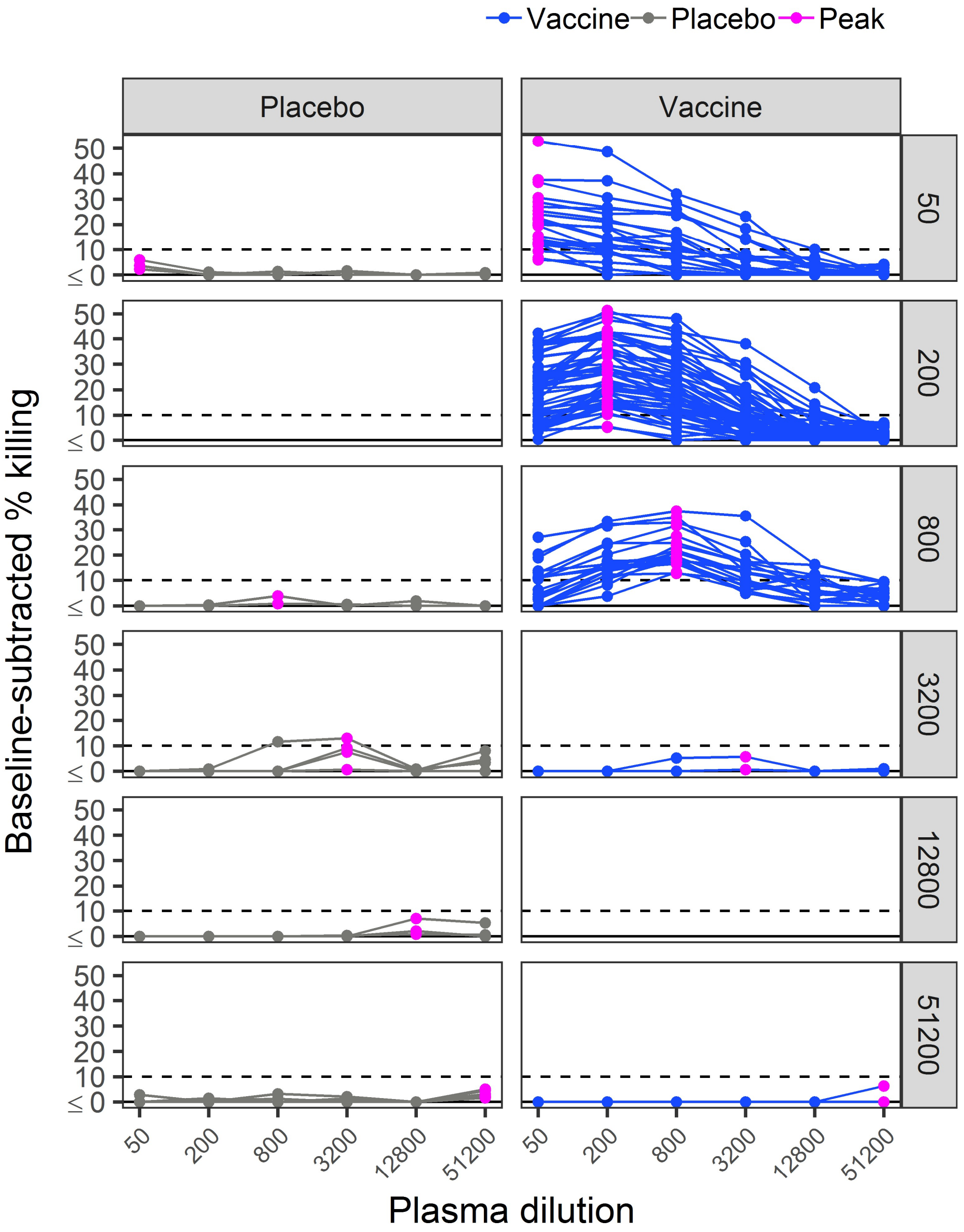

Figure S1. Baseline-subtracted %killing ADCC dilution curves plotted by the dilution at which the peak response is observed and by vaccination group for all IMCs combined.

Partial area under the dilution curve (pAUC)

To determine which dilutions should be included in the computation of the partial area under the baseline-subtracted dilution curve (pAUC), we compare the dilution-specific distribution of baseline-subtracted ADCC responses by vaccine group in Figure S2. We observe clear differences in the distribution between placebo and vaccine recipients in the first four dilutions, while there is clear overlap in distributions of placebos and vaccine recipients at the two dilutions. Since responses at the last two dilutions are primarily noise, they are not included in the area under the curve calculation.

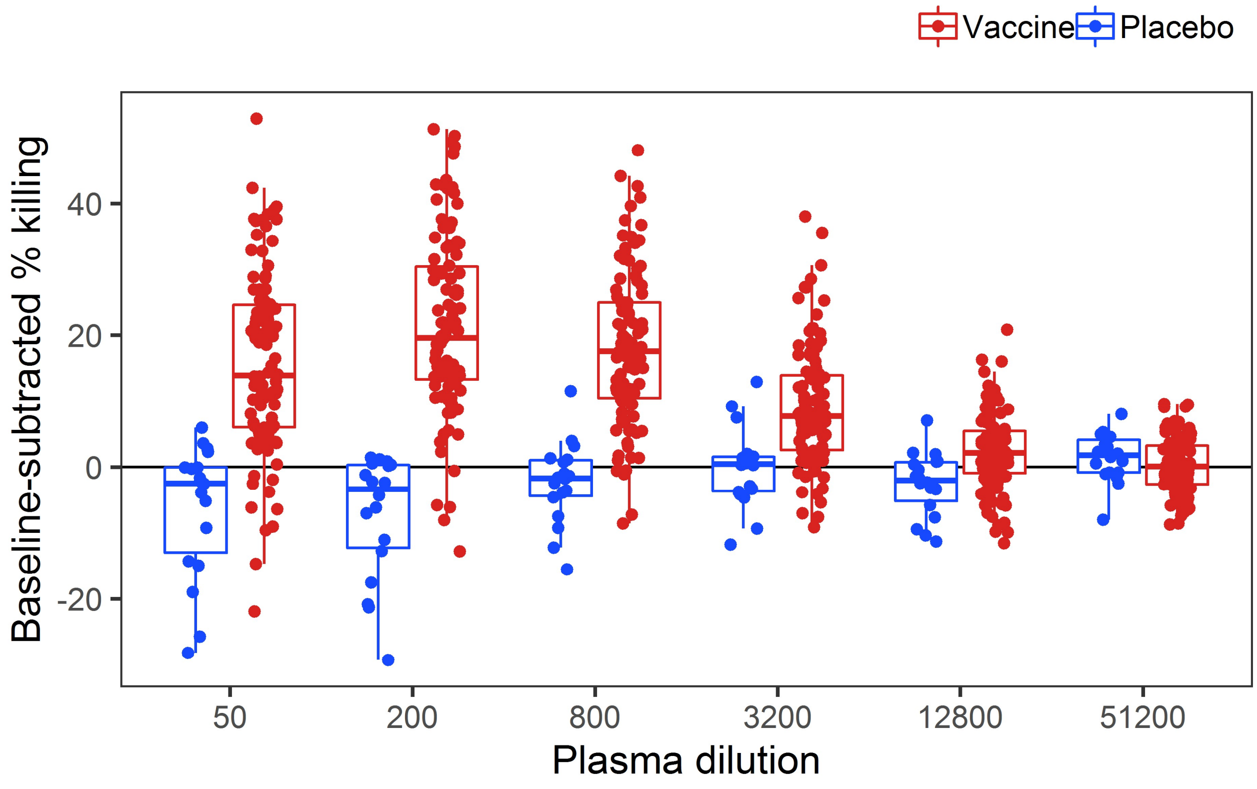

Figure S2: Distribution of baseline-subtracted % killing by dilution and vaccine group for all three IMCs tested.

Additional independent replicates of the dilution curves shown in Figure 1

The dilution curves shown in Figure 1 in the main manuscript were repeated in two additional independent experiments, in which we tested more dilutions so that plasma from the seropositive sample would reach endpoint. Thus, we have results from 3 independent experiments for the first six plasma dilutions, and two independent experiments for the last two plasma dilutions. Each independent experiment included two technical replicates; average % killing is reported. The results are summarized in Figure S3, where we plot the average % killing across all three of the independent experiments and include error bars for 1 standard deviation.

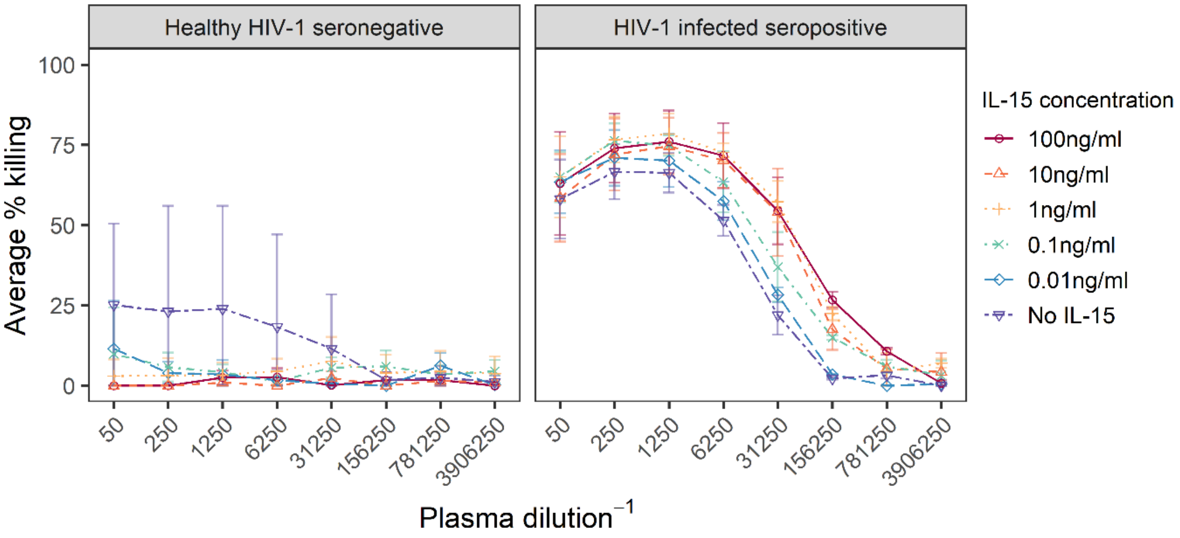

Figure S3. Dilution curves representing the average % specific killing in ADCC assays performed with effector cells treated overnight with IL-15 at the indicated concentrations and plasma from a healthy HIV-1 seronegative or an HIV-1 infected seropositive individual. IL-15 concentration levels below 1 ng/ml are shown in varying shades of blue, while higher concentrations are in oranges and reds. Each point represents the average % killing from 3

independent experiments with error bars of 1 SD for the first 6 plasma dilutions, and 2 independent experiments for plasma dilutions greater than 156250.

Pilot study to determine the dilution scheme for detecting vaccine-induced ADCC activity

The purpose of the pilot study was to determine what dilution scheme could capture complete dilution curves for vaccine recipients while potentially yielding better dilution curves for placebo recipients.

Using samples from 15 HVTN 100 participants (n= 6 placebo recipients and n = 9 vaccine recipients), 2-, 3-, and 4-fold dilution curves were plotted for ADCC responses measured in the presence of IL-15. The average baseline-subtracted percent killing in responders and non-responders for each of the four sets of assay conditions is shown in Figure S4. For samples from vaccine responders, the 2- and 3-fold dilution curves were not sufficient to capture the entire response curve and compute a titer. In fact, the titer could be determined for only 20% of the 5 positive two-fold dilution curves, and 60% of the 5 positive curves from the 3-fold dilution. In contrast, a titer was able to be determined for all positive 4-fold dilution curves. From this small study, we concluded that the 2- and 3-fold dilution series would not be appropriate for capturing complete dilution curves for HVTN studies.

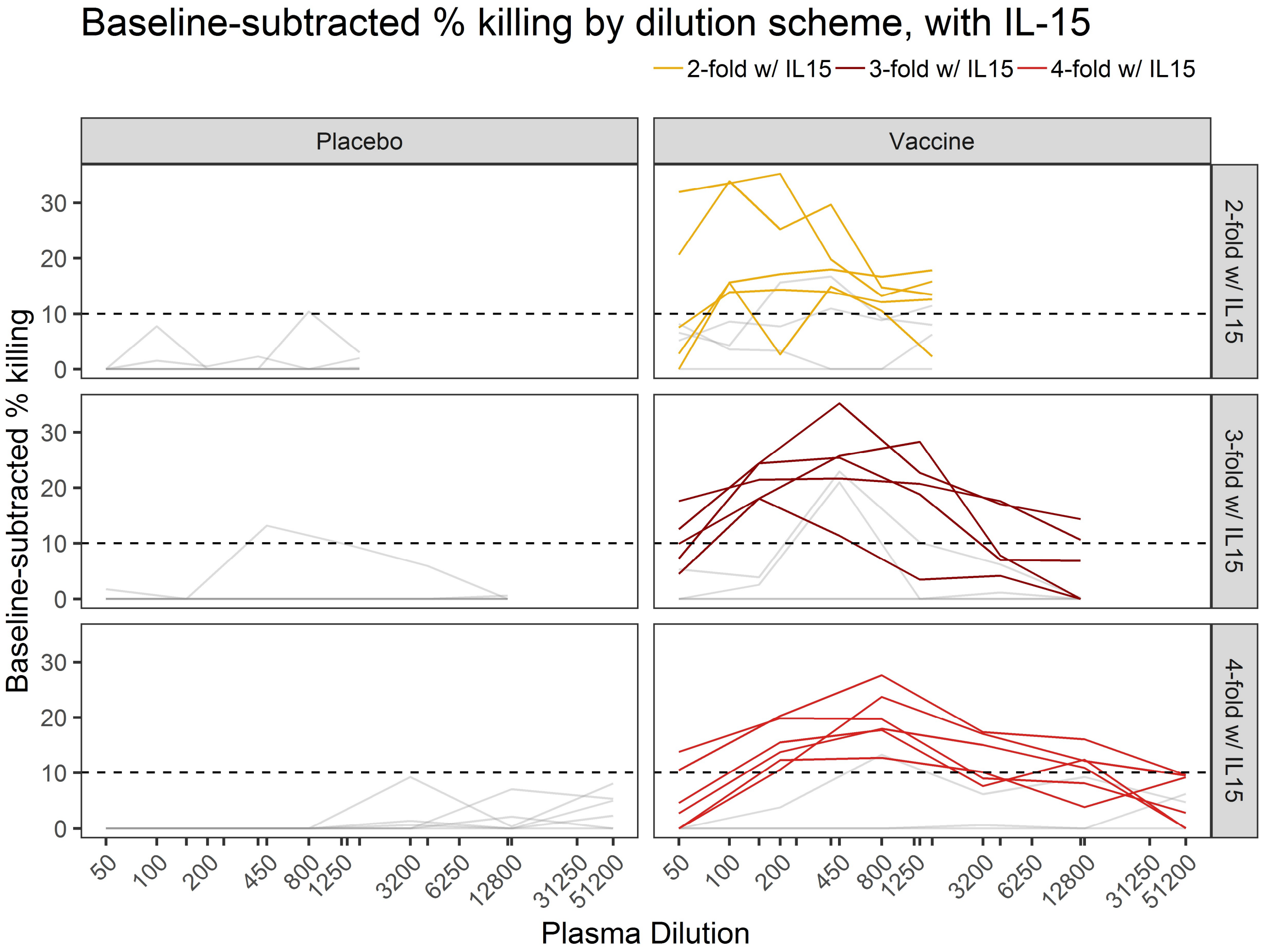

Figure S4. Comparison of average baseline-subtracted ADCC response dilution curves for HVTN 100 responders and non-responders. The assay used target cells infected with the TV1 IMC. All tests were performed in the presence of IL-15.
